# Descriptive, Hospital-Based, 10-Year Study of Malaria Transmission in Goa, a Southwest Indian State in the Malaria Elimination Phase

**DOI:** 10.4269/ajtmh.23-0828

**Published:** 2024-05-07

**Authors:** Rimi Chakrabarti, Kevin Gillespie, Jayashri T. Walke, Mezia Fernandes, Anvily Almeida, Laura Chery-Karschney, Usheer Kanjee, Kristen M. Skillman, John White, Prasad H. Babar, Ligia Pereira, Anjali Mascarenhas, Marina Vaz, Anar Khandeparkar, Edwin Gomes, Holly Janes, Pradipsinh K. Rathod, Manoj T. Duraisingh

**Affiliations:** ^1^ Department of Chemistry, University of Washington, Seattle, Washington;; ^2^ Department of Medicine, Goa Medical College and Hospital, Bambolim, Goa;; ^3^ Fred Hutchinson Cancer Research Center, Seattle, Washington;; ^4^ Department of Immunology and Infectious Diseases, Harvard T. H. Chan School of Public Health, Boston, Massachusetts

## Abstract

The South Asia International Center of Excellence for Malaria Research, an NIH-funded collaborative program, investigated the epidemiology of malaria in the Indian state of Goa through health facility–based data collected from the Goa Medical College and Hospital (GMC), the state’s largest tertiary healthcare facility, between 2012 and 2021. Our study investigated region-specific spatial and temporal patterns of malaria transmission in Goa and the factors driving such patterns. Over the past decade, the number of malaria cases, inpatients, and deaths at the GMC decreased significantly after a peak in 2014–2015. However, the proportion of severe malaria cases increased over the study period. Also, a trend of decreasing average parasitemia and increasing average gametocyte density suggests a shift toward submicroscopic infections and an increase in transmission commitment characteristic of low-transmission regions. Although transmission occurred throughout the year, 75% of the cases occurred between June and December, overlapping with the monsoon (June–October), which featured rainfall above yearly average, minimal diurnal temperature variation, and high relative humidity. Sociodemographic factors also had a significant association with malaria cases, with cases being more frequent in the 15–50-year-old age group, men, construction workers, and people living in urban areas within the GMC catchment region. Our environmental model of malaria transmission projects almost negligible transmission at the beginning of 2025 (annual parasitic index: 0.0095, 95% CI: 0.0075–0.0114) if the current control measures continue undisrupted.

## INTRODUCTION

Malaria has been a consistent feature in Goa, India’s smallest state on the country’s southwest coast, with the entire population living in an endemic region. With 3.3 million cases in 2022, India still bears the burden of 66% of malaria cases in the WHO Southeast Asia region.^1^ However, between 2000 and 2021 the rate of new infections (annual parasitic index [API]) decreased from 19.8 to 2.5 per 1,000 population at risk.^1^ Malaria cases in Goa have followed national trends.^2^ Globally, India is in the “very low” category of the malaria transmission intensity continuum.^3^ Within India, Goa is in the “low-transmission” category with an average API of 0.3 (2014*–*2021), accounting for 0.1% of the country’s malaria caseload^2^ (Supplemental Figure 1). This API places Goa in the National Malaria Control Program’s (NMCP’s) category 1, or malaria elimination phase,^4^ geared toward complete elimination by 2030.^5^ Malaria intervention policies in this category stipulate heightened surveillance with passive case detection (PCD) at all levels of the health system, active case detection to fill the gaps in PCD, case investigation, and identification of areas with residual transmission for targeted interventions.^4^^,^^6^

Since 2012, the Malaria Evolution in South Asia International Center of Excellence for Malaria Research (MESA-ICEMR), an NIH-funded collaborative program,^7^ has studied the epidemiology of malaria in Goa through health facility–based enrollment and data collected at the Goa Medical College and Hospital (GMC).^7^ The GMC is the largest tertiary healthcare facility in the state, located centrally near the capital city of Panjim. This location, combined with the hospital’s policy to provide free or low-cost primary care to Goa residents, makes it the preferred healthcare destination in this state. Although this study is limited to malaria transmission in the GMC catchment region, several factors, including the total number of malaria patients treated by the GMC per year and a catchment region covering nearly the entire state of Goa, provide a high level of confidence in extrapolating the hospital-based data to deduce the status of malaria transmission in Goa. Our study found region-specific spatial and temporal patterns of malaria transmission in the GMC catchment region and the factors driving such patterns. We also observed that malaria cases in the GMC catchment region were clustering in specific regions or hotspots, which could provide insights to local public health agencies about optimal allocations of malaria control resources at the best time and place. Passive case detection via health facility–based surveys with basic geo-components such as this can complement cohort studies to investigate the last remaining foci of transmission in Goa’s rapidly diminishing malaria transmission scenario. This study also puts the Goa findings in the context of the changing epidemiology of malaria in response to intense control measures in other low-transmission, pre-elimination settings globally.

## MATERIALS AND METHODS

### Hospital-based surveillance.

**Figure 1.**
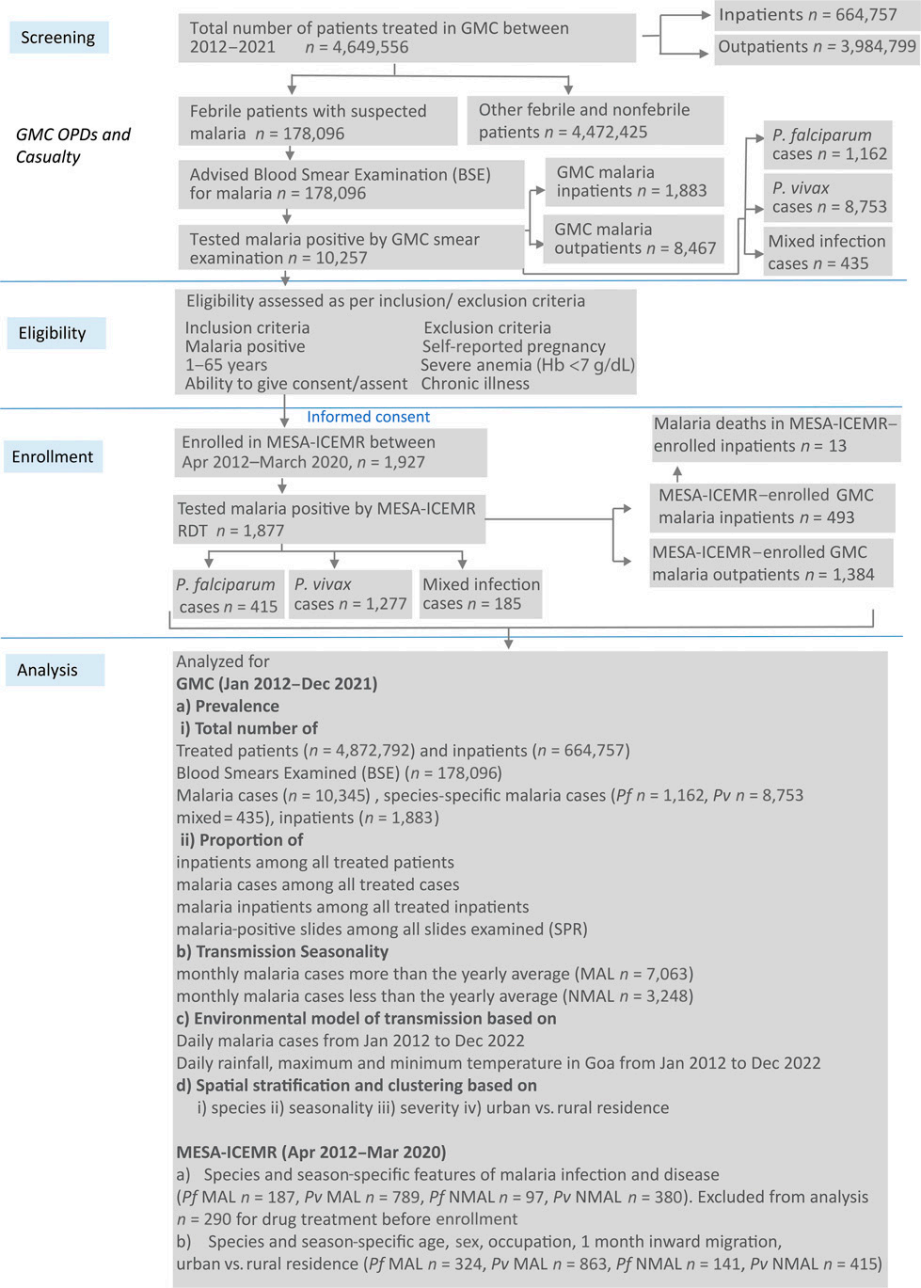
Outline of the GMC-based Goa malaria transmission study. GMC = Goa Medical College and Hospital; Hb = hemoglobin; MESA-ICEMR = Malaria Evolution in South Asia International Center of Excellence for Malaria Research; MAL = malaria season; NMAL = non–malaria season; OPD = outpatient department; *Pf*/*P. falciparum* = *Plasmodium falciparum*; *Pv*/*P. vivax* = *Plasmodium vivax*; RDT = rapid diagnostic test; SPR = slide positivity rate.

This study was conducted between January 2012 and December 2021 with human subjects’ ethics approval from the University of Washington Institute Review Board, the Government of India Health Ministry Screening Committee, and the GMC Institute Ethics Committee. We screened care-seeking febrile individuals in GMC outpatient departments and casualty who were advised to undergo a malaria smear test by the attending physician (Figure 1). Patients aged 1–65 years diagnosed as malaria positive by the GMC smear test were approached for informed consent, excluding pregnant and anemic patients as well as those suffering from chronic diseases. Patients who provided informed consent were enrolled, and a venous blood sample was drawn prior to the start of the antimalarial regimen. All the patients were assessed for disease severity by the attending physician and categorized as having uncomplicated or severe malaria. Study subjects were assigned a unique identification number upon enrollment, and their age, sex, occupation, past travel, and current residential address were noted. All data and collected samples were linked to individual participants through this identification number to maintain subject confidentiality. The current residential address was used to geolocate the anonymized study subjects at the village level.

Blood samples were retested with a rapid diagnostic test (RDT; FalciVax, Zephyr Technologies, Mangalore, India) at the MESA-ICEMR laboratory to confirm malaria positivity and infecting species. Giemsa-stained thick and thin smears of the samples were prepared and examined for parasitological parameters. A subset of the enrolled samples, selected at random, was adapted in a laboratory culture system, and an average parasite multiplication rate was determined from the growth rate of parasites during adaptation. For statistical analysis, malaria cases were stratified by 1) the *Plasmodium* species causing infection (i.e., *Plasmodium falciparum* or *Plasmodium vivax* or a mixed infection) and 2) the disease severity (i.e., uncomplicated or severe). A third stratification was used to determine seasonality of malaria transmission. We defined malaria season (MAL) as the months of a given year in which the monthly number of cases exceeded the average annual number of cases at the GMC. The remaining months were designated as non–malaria season (NMAL). Seasonality was thus determined for each individual year of the 10-year study period. We compared infection and disease parameters (listed in Table 1) by nonparametric unpaired *t*-test (Mann-Whitney) in GraphPad Prism (v. 9; GraphPad Software, Boston, MA). Infection rates were compared between groups of individuals defined on the basis of seasonal/nonseasonal malaria using two-sided 0.05-level χ^2^ tests. The Bonferroni method was used to correct for multiple comparisons.

**Table 1:** Characteristics of seasonal malaria infections and the resulting disease in MESA-ICEMR–enrolled samples

Parameters	Species	NMAL					MAL					*P*-Value
		*n* (%)	Median	IQR	Range	Mean	*n* (%)	Median	IQR	Range	Mean	
Total enrolled cases	Pf	92	–	–	–	–	223	–	–	–	–	–
	Pv	343	–	–	–	–	841	–	–	–	–	–
Infection												
Parasitemia (%)	Pf	92	0.46	0.15–1.35	0–9.2	1.13	220	0.64	0.22–1.85	0–24.65	1.63	0.08
	Pv	302	0.13	0.04–0.4	0–2.06	0.31	770	0.2	0.06–0.62	0–6.52	0.48	0.0002
Parasite density (per μl)	Pf	89	4,720	809.5–14,265	0–115,368	10,734	219	4,470	1,249–13,440	0–224,960	14,972	0.87
	Pv	302	2,160	796.3–6,110	0–29,520	4,533	771	2,048	666–5,928	0–185,739	5,515	0.54
Gametocytemia (%)	Pf	89	0	0	0–0.5	0.019	219	0	0	0–0.49	0.012	0.42
	Pv	302	0	0	0–0.37	0.012	770	0	0	0–0.75	0.023	0.68
Gametocyte density (per μl)	Pf	90	0	0	0–1,480	87.53	218	0	0	0–4,367	171.2	0.79
	Pv	302	0	0–259.5	0–4,536	253.2	772	0	0–277.5	0–7,240	267.5	0.82
Gametocyte-carrying samples	Pf	18 (18.6)	–	–	–	–	30 (16.3)	–	–	–	–	–
	Pv	142 (34.2)	–	–	–	–	310 (35.9)	–	–	–	–	–
iRBC fraction producing gametocytes (%)	Pf	92	0	0	0–33	1.32	214	0	0	0–36	0.75	0.28
	Pv	316	0	0	0–66.67	3.55	771	0	0	0–78	3.54	0.56
PMR	Pf	119	4.12	2–5.8	0–17.3	4.33	287	4.93	2.9–7	0–30.7	5.36	0.013
Disease												
Severity score	Pf	84	0	0	0–3	0.25	210	0	0	0–7	0.65	0.02
	Pv	331	0	0	0–7	0.12	818	0	0	0–7	0.06	0.27
Caseload of severe patients	Pf	21 (22.8)	–	–	–	–	53 (23.7)	–	–	–	–	–
	Pv	20 (5.8)	–	–	–	–	37 (4.4)	–	–	–	–	–
Temperature at enrollment (°C)	Pf	89	101.1	99.25–103.4	96.3–107.2	101.3	221	100.2	98.7–102.7	94.6–107.6	100.8	0.09
	Pv	343	100.5	98.8–103	95.9–107.2	101	839	100.2	94–102.5	94–107.6	100.7	0.11
Hematocrit at enrollment (%)	Pf	82	34.75	28.5–38.5	14.5–52.5	33.52	170	33.5	28.48–39	13–65.65	33.78	0.93
	Pv	297	35	29–39.75	14–51.5	34.26	745	35.5	30.5–40.5	9.5–70.5	35.31	0.09
Age (years)	Pf	92	24	20–30	3–57	26.77	223	24	20–34	3–60	26.91	0.58
	Pv	343	25	20–34	1–65	28.17	841	25	20–31	2–66	27.21	0.06

Malaria cases that were treated with antimalarials before enrollment were excluded from this analysis. MESA-ICEMR = Malaria Evolution in South Asia International Center of Excellence for Malaria Research; iRBC = infected red blood cells; IQR = interquartile range; MAL = malaria season; NMAL = non–malaria season; Pf *= Plasmodium falciparum*; PMR = parasite multiplication rate; Pv *= Plasmodium vivax*.

### Environmental model of malaria transmission.

Daily rainfall (0.25° × 0.25°), temperature (1° × 1°), and relative humidity (0.25° × 0.25°) data were obtained from the open-source gridded data repository of the Indian Meteorology Department^8^^,^^9^ and the European Center for Medium-Range Weather Forecasts, ERA5 Copernicus Climate Data Store.^10^^,^^11^ Malaria cases were confirmed by any or all of the following - a positive RDT, a positive identification on a thick or thin blood smear, and positive replication using quantitative polymerase chain reaction. Infection rates were calculated by dividing the cumulative number of monthly cases by the census population size of Goa, and its value was scaled per 1,000 persons. We modeled monthly cumulative cases or monthly infection rates using the following covariates summarized from the daily environmental data: cumulative monthly rainfall, average monthly relative humidity, and the difference between the maximum and minimum monthly temperatures. We generated a negative-binomial generalized linear model with a log link function for monthly cases and a Gaussian generalized linear model with an inverse gamma link function for monthly infection rates.

For seasonal cumulative monthly cases or infection rate trends, the model is simplified below:

$$y_{i}∼\beta_{0}+\beta_{1}r_{i}+\beta_{2}h_{i}+\beta_{3}c_{i},$$

where *y_i_* is the outcome (cumulative monthly cases or infection rate), *r_i_* is the cumulative monthly rainfall, *h_i_* is the average monthly relative humidity, *c_i_* is the difference between the maximum and minimum monthly temperatures, and *i* represents each month-year from January 2012 to December 2021.

To assess case or infection rate trends across years, we used the model simplified below:

$$y_{i}∼\beta_{0}+\beta_{1}r_{i}+\beta_{2}h_{i}+\beta_{3}t_{i}+\overset{1}{\underset{j=4}{\sum }}\beta_{j}\cdot C_{j}\left(t_{i}\right),$$

where *y_i_* is the outcome (cumulative monthly cases or infection rate), *r_i_* is the logarithm of cumulative monthly rainfall, *h_i_* is the average monthly relative humidity, *t_i_* is the number of months since December 2012, and *i* represents each month in the dataset from January 2012 to December 2021. The additional time covariate was included in this model based on months since December 2012 to assess the temporal trend of monthly malaria cases and infection rates. Time was modeled using a spline with two inflection points, approximating a quadratic curve fit to the trend of the monthly cases or infection rate. Using a spline approximation of a quadratic equation for time is a limitation of our current modeling approach because it excludes the possibility of the data trending back up in the future. However, given the trend of the collected data and the intensifying interest in Goa to eliminate malaria infection, this was not a major concern.

The model was fit to the data in two ways: 1) using the full set of data and 2) excluding data after the COVID-19 lockdowns to assess the sensitivity of the model to possible erroneous low counts.

### Spatial analysis.

We analyzed the spatial pattern of GMC-treated malaria cases using ArcGIS Pro (v. 3.0.2, ESRI, Redlands, CA). Patient addresses were used to generate both spatial point data and village-level spatial aggregate data. The first level of stratification involved mapping village-level aggregate data yearly, followed by a spatial autocorrelation analysis for local clustering and hotspot analysis. Anselin Local Moran’s I and Getis Ord GI* analyses were used according to ArcGIS best practice guidelines^12^^–^^14^ to identify statistically significant hotspots, cold spots, and spatial outliers. The maximum distance at which each spatial feature (village in our case) had at least eight neighboring features was calculated and used in the analyses to keep the scale of analysis constant throughout the study area. We defined a hotspot as a spatial feature that had a statistically significant higher number of malaria cases compared with the average level in the neighborhood features. The Anselin Local Moran’s I index determined whether spatial features were part of a cluster or outliers. The *z* score and *P*-values in this analysis indicated the statistical significance (set at the 95% CI) of such classification. The output was depicted as clusters of high values (high-high), clusters of low values (low-low), outliers with a high-value feature predominantly surrounded by low-value features (high-low), and vice versa (low-high).^13^ The Getis Ord Gi* local statistic defined a statistically significant hotspot as a feature of high value that was surrounded by other features of high value. It is a *z* score itself, and along with the *P*-value it depicted the statistically significant clustering of high values as hotspots and low values as cold spots.^14^ We further stratified malaria transmission hotspots by 1) *Plasmodium* species: *P. falciparum* and *P. vivax*, 2) season: MAL and NMAL, 3) disease severity: uncomplicated and severe, 4) residence in urban versus rural region, 5) travel versus no travel to Goa in the last 1 month, and 6) time (2012–2021).

## RESULTS

### Malaria transmission rate in the GMC catchment region has decreased over 80% in the last 10 years.

**Figure 2.**
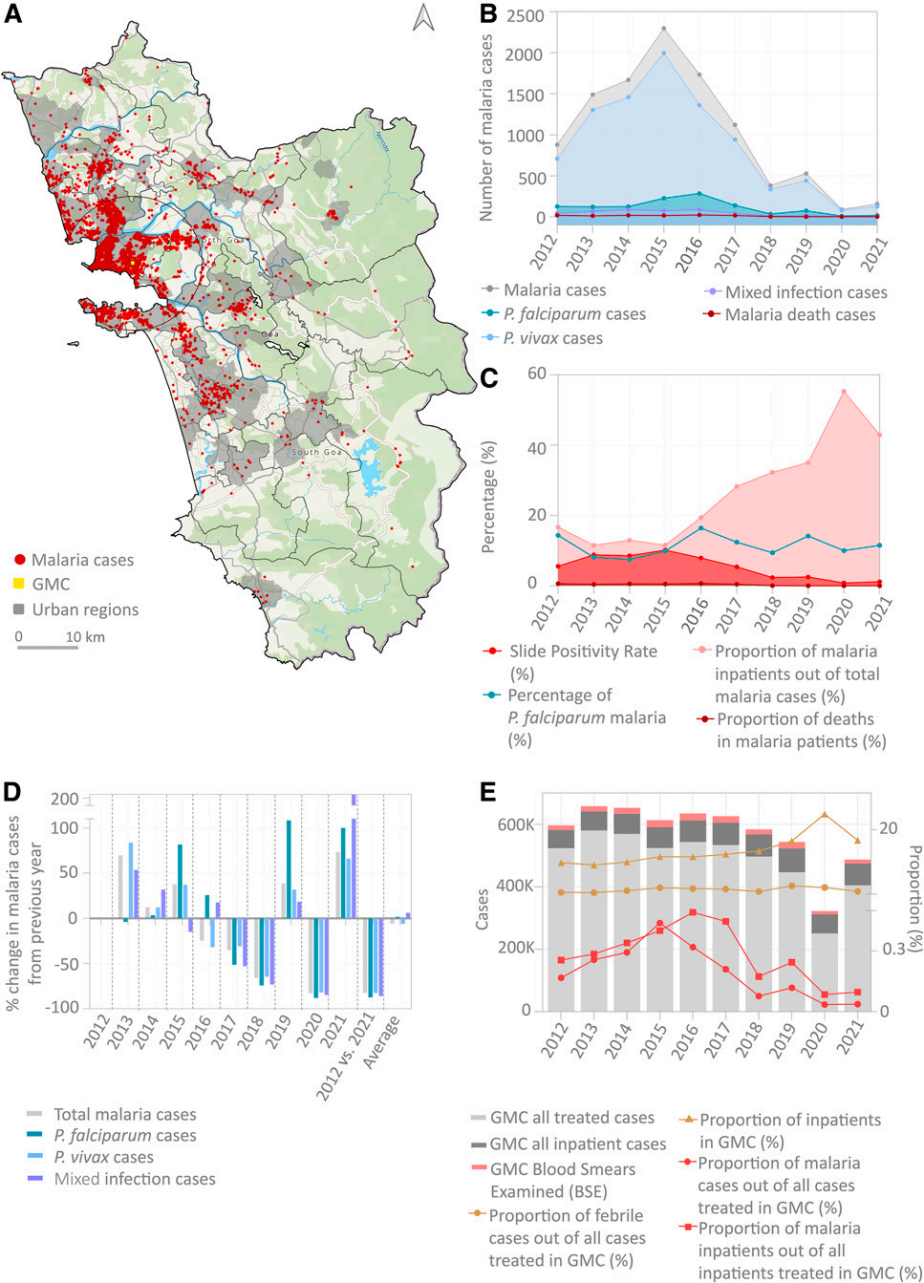
Data from malaria cases treated in GMC and enrolled by MESA-ICEMR gives a snapshot of malaria transmission in Goa. (**A**) The GMC catchment area and distribution of GMC cases in the urban and rural regions of Goa; 80% of malaria cases treated in the GMC came from within a 10-km radius (red circles). Internal boundaries enclose the primary healthcare center regions superimposed on the topographic map of Goa. (**B**) Malaria incidence: Total number of malaria cases, cases caused by *Plasmodium falciparum, Plasmodium vivax*, and mixed infection; total number of death cases due to malaria. (**C**) Proportional malaria incidence: Slide positivity rate (%), proportion of inpatients, and proportion of deaths among total number of malaria cases (%). (**D**) Change in transmission: Percentage change in number of malaria cases compared with previous year. (**E**) Proportion of febrile and malaria cases out of all cases treated in the GMC. GMC = Goa Medical College and Hospital; MESA-ICEMR = Malaria Evolution in South Asia International Center of Excellence for Malaria Research; *P. falciparum* = *Plasmodium falciparum*; *P. vivax = Plasmodium vivax*.

The data we collected at the GMC through the MESA-ICEMR program between 2012 and 2021 provided a description of malaria transmission in Goa (Figure 2). In these 10 years, the GMC treated 10,341 malaria patients (Supplemental Table 1) from all over Goa (Figure 2A), suggesting that this hospital is a statewide catchment region. Approximately 80% of the patients resided within a 10-km radius of the hospital (Figure 2A) and were from the neighboring areas of Bambolim, Panjim, Dona Paula, Ribander, Old Goa, Goa Velha, Vasco Da Gama, and Cortalim (Supplemental Figure 2). The number of malaria cases treated by the GMC declined after a peak in 2015 (Figure 2B; Supplemental Figure 3). This reduction was observed across *P. falciparum*, *P*. *vivax*, and mixed infections. This overall shift is also reflected in the malaria parameters tracking relative proportions of cases (Figure 2C). The slide positivity rate (SPR) has been steadily declining since 2014. The proportion of malaria cases caused by *P. falciparum* has remained consistent, with intermittent fluctuations. However, the proportion of inpatient malaria cases at the GMC has steadily increased over the years (Supplemental Table 1). There was an 82% decrease in total malaria cases in 2021 compared with 2012 (Figure 2D), with a greater decrease in *P. falciparum* cases (86%) than in *P. vivax* cases (81%). The MESA-ICEMR data from enrolled patients during this time also showed a decrease in mean parasitemia from 2% to 0.35%, whereas mean gametocyte density increased from 146 to 385 per microliter (exception 2012) (Supplemental Table 1).

Every year, the GMC treats approximately 500,000 patients (Figure 2E), of whom approximately 66,000 (14%) are treated as inpatients. This figure has remained relatively consistent since 2012. Admissions in 2020 were a notable exception, which could be attributed to disruptions in health-seeking behavior or data reporting during the first year of the COVID-19 pandemic. On average, 17,000 febrile patients have been malaria smear tested every year in the GMC, accounting for 4% of all patients treated in the GMC. Of these, approximately 1,000 febrile cases tested positive for malaria per year, contributing to 0.2% of the total cases treated in the GMC. This proportion dropped from a peak of 0.4% in 2014 to 0.08% in 2021. Thus, the absolute and relative numbers of malaria cases have been steadily declining, whereas the total number of patients (outpatient or inpatient) and febrile patients treated at the GMC has remained relatively consistent.

The broad pattern of decreasing GMC malaria cases is consistent with that observed in the state (Supplemental Figure 2A–D), suggesting that the GMC data are a reliable sample of malaria transmission in Goa.

### Malaria transmission in the GMC catchment region is perennial and cyclical with seasonal peaks and troughs.

**Figure 3.**
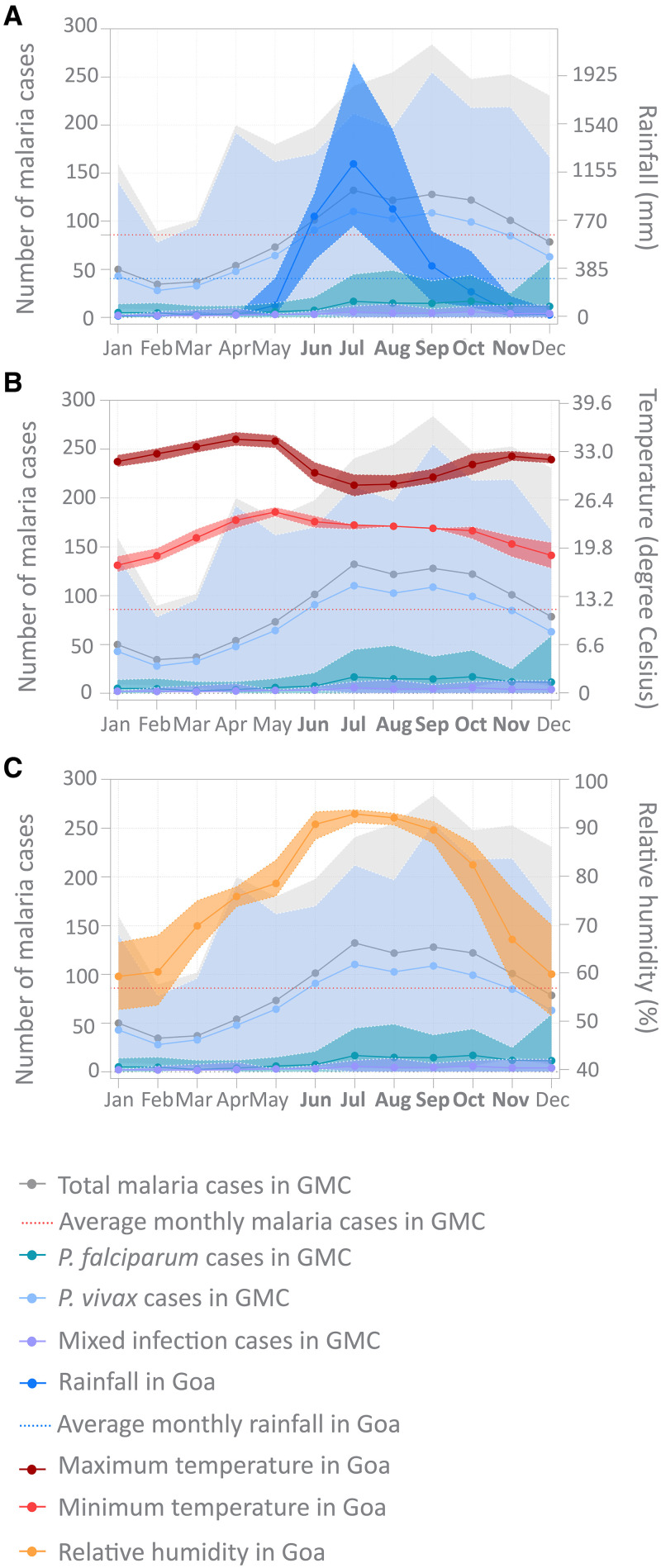
Seasonal transmission of malaria in Goa. (**A–C**) Association of rainfall (**A**), temperature (**B**), and relative humidity (**C**) with number of malaria cases in the GMC during different months of the year between 2012 and 2021. Closed circles with connecting lines represent monthly mean value over the course of 10 years. The shaded regions represent the range during this time period. The red dotted line represents the monthly average number of malaria cases between 2012 and 2021, and the blue dotted line represents monthly average rainfall. Together, they demarcate the months (in bold) with a more-than-average number of malaria cases and rainfall (MAL). GMC = Goa Medical College and Hospital; MAL = malaria season; *P. falciparum* = *Plasmodium falciparum*; *P. vivax = Plasmodium vivax.*

We examined the GMC dataset to determine the seasonal pattern of malaria transmission in Goa and the characteristics of seasonal infections (Figure 3; Tables 1 and 2; Supplemental Figure 3). *Plasmodium vivax* MAL transmission occurred between June and November, accounting for 68% of total *P. vivax* cases, which peaked first in July and then in September. *Plasmodium falciparum* MAL transmission occurred between July and December, accounting for 75% of *P. falciparum* cases, which peaked in July and September and then decreased from December onward (Figure 3; Supplemental Figure 3). Malaria season transmission in general was most common during the monsoon (June–October), with higher-than-yearly average rainfall (484.5 mm, 95% CI: 357–612 mm), minimum diurnal variation (difference between daily maximum and minimum) in temperature (8.3°C, 95% CI: 7.6–9°C), and high relative humidity (87.4%, 95% CI: 79.8–85.8%) (Table 1). Severe cases of malaria followed a similar pattern of transmission and transmission peak. Transmission occurred throughout the year, even during the dry season with little or no rainfall. However, the highest transmission (top 10%) happened when the monthly average rainfall exceeded a minimum threshold of 23.3 mm, the temperature ranged between 22.8°C and 30°C, and relative humidity was between 72% and 93% (Table 2). We also noted interannual variation in relative intensity, timing of onset, and extent of MAL and NMAL that happened around the average pattern (Supplemental Figure 3). Malaria season also occurred during the seasonal peak (May–October) of the dominant vector in this state, *Anopheles stephensi*.^15^ Non–malaria season occurred during the drier months (January–May) with little or no rainfall and was sustained by the supporting vector *Anopheles subpictus*.^15^

**Table 2:** Malaria cases in the GMC in relation to rainfall, temperature, and humidity in Goa

Environmental Parameters	Total Malaria Cases				*Plasmodium falciparum* Malaria Cases				*Plasmodium vivax* Malaria Cases			
	Less Than Yearly Average	More Than Yearly Average	Bottom 90th Percentile	Top 10th Percentile	Less Than Yearly Average	More Than Yearly Average	Bottom 90th Percentile	Top 10th Percentile	Less Than Yearly Average	More Than Yearly Average	Bottom 90th Percentile	Top 10th Percentile
Cumulative rainfall (mm)												
Range	0–38.3	0.1–2,036	0–2,036	3.5–1,003	0–1,499	0–2,036	0–1,499	0.1–2,036	0–1,463	0–2,036	0–2,036	3.5–1,006
Mean	138.2	486.9	272.7	590.2	167.1	494.8	262.5	682	135.8	504.2	273.4	612.3
Median	4.9	346.7	23.3	635.8	8.3	427.8	24.1	635.8	5.5	428.1	23.7	663.9
SD	331.2	461.5	447.4	295.9	381.9	457	408	582.1	355.9	457.5	445.4	299.8
95% CI	54.8–221.6	364.4–609.3	187–358.5	402–778	75.3–258.8	364.9–624.7	184.3–340.6	312.1–1,052	45.4–226.2	375.6–632.9	188.5–358.4	411–811.7
Temperature maximum (°C)												
Range	27.8–35.7	26.9–35.2	26.9–35.7	28.4–33.3	27.8–35.7	26.9–35.2	27.8–35.7	26.9–32.5	27.8–35.2	26.9–35.2	26.9–35.7	28.4–33.3
Mean	32.5	30.7	31.7	29.8	32.3	30.48	31.7	29.5	32.2	30.47	31.7	29.8
Median	32.7	30.3	32.0	29.4	32.5	30	31.9	29.45	32.4	30	31.8	29.4
SD	1.9	2.2	2.1	1.3	1.8	2	2	1.6	1.7	2	2.1	1.39
95% CI	32–32.9	30.1–31.2	31.3–32.1	28.9–30.6	31.8–32.7	29.8–31	31.3–32.1	28.5–30.6	31.8–32.6	29.9–31	31.3–32.1	28.9–30.7
Temperature minimum (°C)												
Range	16.6–25.4	18.7–25.2	16.6–25.4	21.4–24.2	16.6–25.4	17.5–24.8	16.6–35.4	18.7–24.2	16.6–25.4	17.5–24.8	16.6–25.4	21.4–24.2
Mean	21.1	22.7	21.5	22.7	21	22.5	21.5	22.4	20.5	22.5	21.5	22.8
Median	21.0	22.7	22.4	22.8	21	22.7	22.4	22.7	20.3	22.7	22.4	22.9
SD	2.6	1.2	2.2	0.7	2.4	1.3	2.2	1.3	2.3	1.3	2.2	0.7
95% CI	20.4–21.8	22.4–23	21.1–21.9	22.3–23.2	20.4–21.6	22.2–22.9	21.1–22	21.5–23.3	19.9–21.1	22.2–22.9	21.1–21.9	22.3–23.3
Temperature difference (°C)												
Range	4.9–15.1	4.5–12.8	4.5–15.1	5.9–11.8	4.9–15.1	4.5–13.8	4.8–15.1	4.5–12.8	4.8–15.1	4.5–13.8	4.5–15.1	5.9–11.8
Mean	11.3	7.9	10.2	7.0	11.3	7.8	10.2	7.1	11.7	7.8	10.1	7
Median	12.0	7.3	10.7	6.5	12	6.9	10.6	6.5	12.3	7	10.6	6.2
SD	2.8	2.2	3.1	1.6	2.7	2.4	3	2.4	2.7	2.4	3.1	1.6
95% CI	10.6–12	7.3–8.5	9.6–10.8	6–8	10.6–12	7.2–8.5	9.6–10.7	5.5–8.7	11–12.4	7.2–8.5	9.5–10.7	5.8–8.1
Relative humidity (%)												
Range	51.1–93	60.8–93.7	51.1–93.7	69.2–92.5	51.1–93.6	60.8–93.7	51.1–93.6	66.3–93.7	51.1–93.6	60.8–93.7	51.1–93.7	69.2–92.5
Mean	70.6	84.4	75.1	88.7	70.7	84.5	75.3	86.9	68.9	84.6	75.29	88.6
Median	69.9	89.6	75.6	90.9	69.6	89.6	75.9	90.75	66.6	89.7	75.75	91.3
SD	11.7	9.3	12.9	6.4	12	9.9	12.9	8.9	11.5	9.8	12.91	6.7
95% CI	67.7–73.6	82–86.9	72.6–77.6	84.6–92.8	67.8–73.6	81.6–87.3	72.8–77.8	81.2–92.5	66–71.8	81.8–87.4	72.8–77.7	84.1–93.1

Bottom 90th percentile and top 10th percentile of the cases denote the bottom 90% transmission and top 10% transmission per year. Thirty days prior meteorological data are considered for this analysis to accommodate development of adult mosquitoes from eggs (10–13 days) and development of malaria parasites in the mosquito midgut (7–14 days). GMC = Goa Medical College and Hospital.

Malaria cases during the MAL and NMAL differed in infection and disease parameters (Table 1). Malaria season infections caused by *P. vivax* had higher average parasitemia, parasite density, gametocytemia, gametocyte density, and proportion of gametocyte-positive samples compared with NMAL *P. vivax* infections. Malaria season infections caused by *P. falciparum* followed a similar pattern except for gametocytemia, which was slightly higher in NMAL than in MAL. The proportion of gametocyte-carrying samples and fraction of infected red blood cells (iRBCs) producing gametocytes were comparable in both seasons. Malaria season and NMAL showed mixed patterns for species-specific severity. Severe *P. falciparum* cases constituted a higher caseload and led to more severe disease manifestation during MAL. Severe *P. vivax* malaria cases had an almost equal caseload and severity spectrum in both seasons. Although the proportion of gametocyte-positive samples and the fraction of iRBCs producing gametocytes were comparable in both seasons, the average gametocyte density was higher in MAL, suggesting that parasites from both species invest more in transmission during this season^16^^–^^18^ to take advantage of higher vector abundance.^15^ In addition, *P. falciparum* virulence was significantly higher in MAL, considering a higher parasite in vitro multiplication rate during adaptation as a proxy indicator. At enrollment, temperature, hematocrit, and age of the malaria patients were also comparable in both seasons.

### Malaria transmission in the GMC catchment region is influenced by sociodemographic factors.

**Figure 4.**
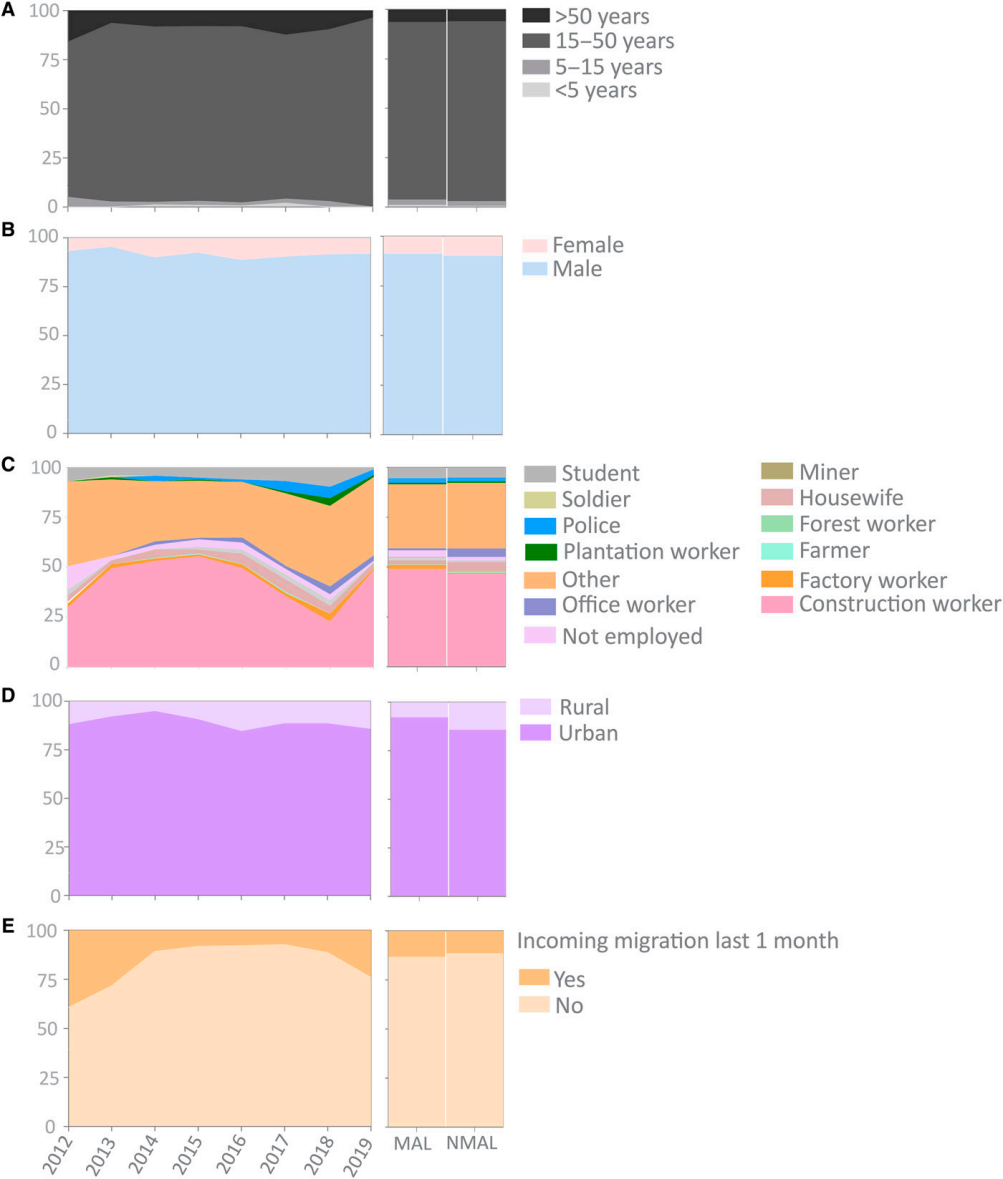
Sociodemographic stratifications of MESA-ICEMR enrolled malaria cases. (**A–D**) Sociodemographic parameters considered here are age (**A**), sex (**B**), occupation (**C**), region of residence (**D**), and inward migration (1 month; **E**). Interannual (2012–2019) and seasonal variations (MAL and NMAL) for each parameter are represented side by side. Sociodemographic data could not be collected in 2020 and 2021 owing to restrictions imposed on patient enrollment that involved face-to-face interaction. MESA-ICEMR = Malaria Evolution in South Asia International Center of Excellence for Malaria Research; MAL = malaria season; NMAL = non–malaria season.

We analyzed the MESA-ICEMR–enrolled subset (Figure 1) to stratify the relative proportions of malaria cases by sociodemographic factors in Goa (Figure 4). The highest number of malaria cases was in the male 15–50-year-old age group irrespective of the season and *Plasmodium* species (Figure 4A). This group also harbored the highest asexual parasite load, with average parasitemia of 0.62% (Supplemental Table 2). Conversely, the above 50-year-old age group had a higher gametocyte load and contained twice the number of gametocyte-positive samples (62%) compared with other age groups (average 30%). The average age of infected patients shifted slightly from 26.2 years in 2013 to 29.3 years in 2019 and was similar for *P. falciparum* (26 years) and *P. vivax* (27 years) malaria (Table 1; Supplemental Table 1). Sex proportion in the enrolled cases was highly skewed, with more than 85% of the infected subjects being male (Figure 4B). More than 50% of these malaria patients self-described their occupation as construction worker (Figure 4C). At least 12.8% of the study subjects had traveled to Goa from other parts of India in the previous month (Figure 4D). This proportion was higher in *P. falciparum* cases (14.6%) than in *P. vivax* cases (11.8%). Approximately 90% of malaria patients resided in designated urban regions of Goa^19^ (Figure 4E). We noted interesting patterns while comparing species-season–specific transmission reservoirs in different occupational groups (Supplemental Table 3). The occupational groups of construction workers, farmers, housewives, plantation workers, and police contributed more to *P. falciparum* transmission in NMAL than in MAL and may be sustaining infections during the dry season. The occupational groups of housewives, local traders, and plantation workers, along with a diverse group of occupations designated as “other,” carried out a similar role for *P. vivax* transmission.

### Spatial stratification of malaria cases in the GMC catchment region reveals a hotspot.

**Figure 5.**
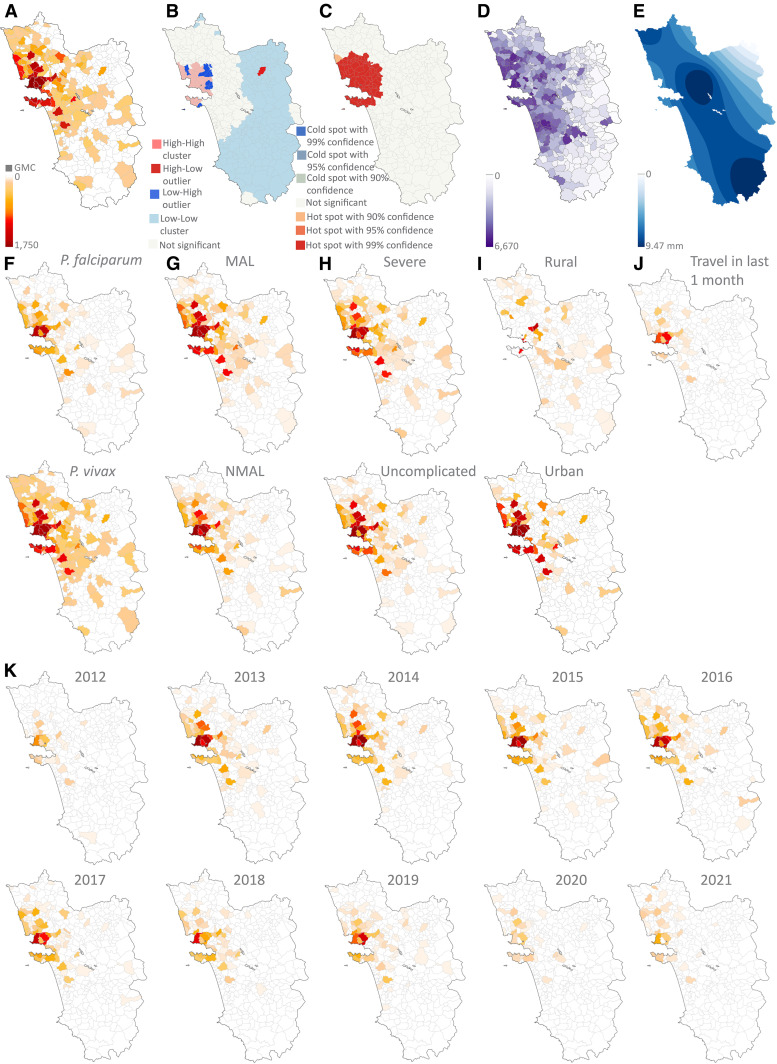
(**A**) Spatial stratification of malaria patients treated in the GMC. (**B** and **C**) Malaria hotspots projected by Anselin Moran’s Local I analysis (**B**) and Getis Ord GI* (**C**) analysis of GMC malaria patients. (**D–K**) The spatial stratification is further categorized by population density (**D**), daily average rainfall (millimeters) (**E**), *Plasmodium* species – *P. falciparum* and *P. vivax* (**F**), malaria seasonality: NMAL and MAL (**G**), malaria severity: uncomplicated and severe (**H**), urban versus rural residence (**I**), travel in the last 1 month (**J**), and time–yearly from 2012 to 2021 (**K**). The internal map borders show the village boundaries. GMC = Goa Medical College and Hospital; MAL = malaria season; NMAL = non–malaria season; *P. falciparum* = *Plasmodium falciparum*; *P. vivax = Plasmodium vivax*.

Malaria patients from 84% of Goa primary healthcare center (PHC) jurisdictions (27 out of 32) and 40% of Goa villages (159 out of 400) were treated in the GMC between 2012 and 2021 (Figure 2A). We analyzed spatial data from the GMC catchment region to determine whether infections were clustered in specific region(s) and if this clustering was influenced by parasite species, seasonality, disease severity, urban versus rural residence, and travel in the last 1 month. Basic spatial stratification indicated several urban regions with higher-than-average malaria cases (Figure 5A). Clustering and hotspot analyses of GMC-treated malaria cases identified a malaria hotspot at the northwest quadrant of Goa (Figure 5B and C). This included the centrally located capital city of Panjim, another prominent city Mapusa in the north, and GMC-adjacent PHC regions of Chimbel, Corlim, Candolim, Porvorim, Aldona, and Maem. It also included the prominent south Goa port city Vasco-Mormugao and the adjacent, mostly urban PHC Cortalim (Supplemental Figure 4A). The Anselin Moran’s I index also identified Valpoi in northeast Goa as a high-low outlier. A similar hotspot pattern was observed for malaria cases when adjusting for population (Supplemental Figure 4B–D). The association between number of malaria cases per village and population density was nonlinear, with a moderate correlation (Spearman ρ = 0.5) (Figure 5D). The hotspot also overlapped with regions receiving moderate rainfall (Figure 5E).

Further stratifications based on parasite species (Figure 5F), transmission season (Figure 5G), severity of disease (Figure 5H), and urban versus rural residence (Figure 5I) showed no specific clustering, confirmed by Getis Ord GI* (not shown). However, malaria patients who had traveled to Goa in the last month were concentrated more in the Panjim and Chimbel PHC regions (Figure 5J). Temporal stratification (Figure 5K) showed a shrinking GMC catchment region in the past 2 years (2020–2021) compared with the high-transmission years (2013–2017). The Getis Ord GI* analysis indicates that the malaria hotspot (Figure 5C) remained unchanged through the study period (not shown).

### Model of malaria transmission in the GMC catchment region predicted a continued downward trend.

**Figure 6.**
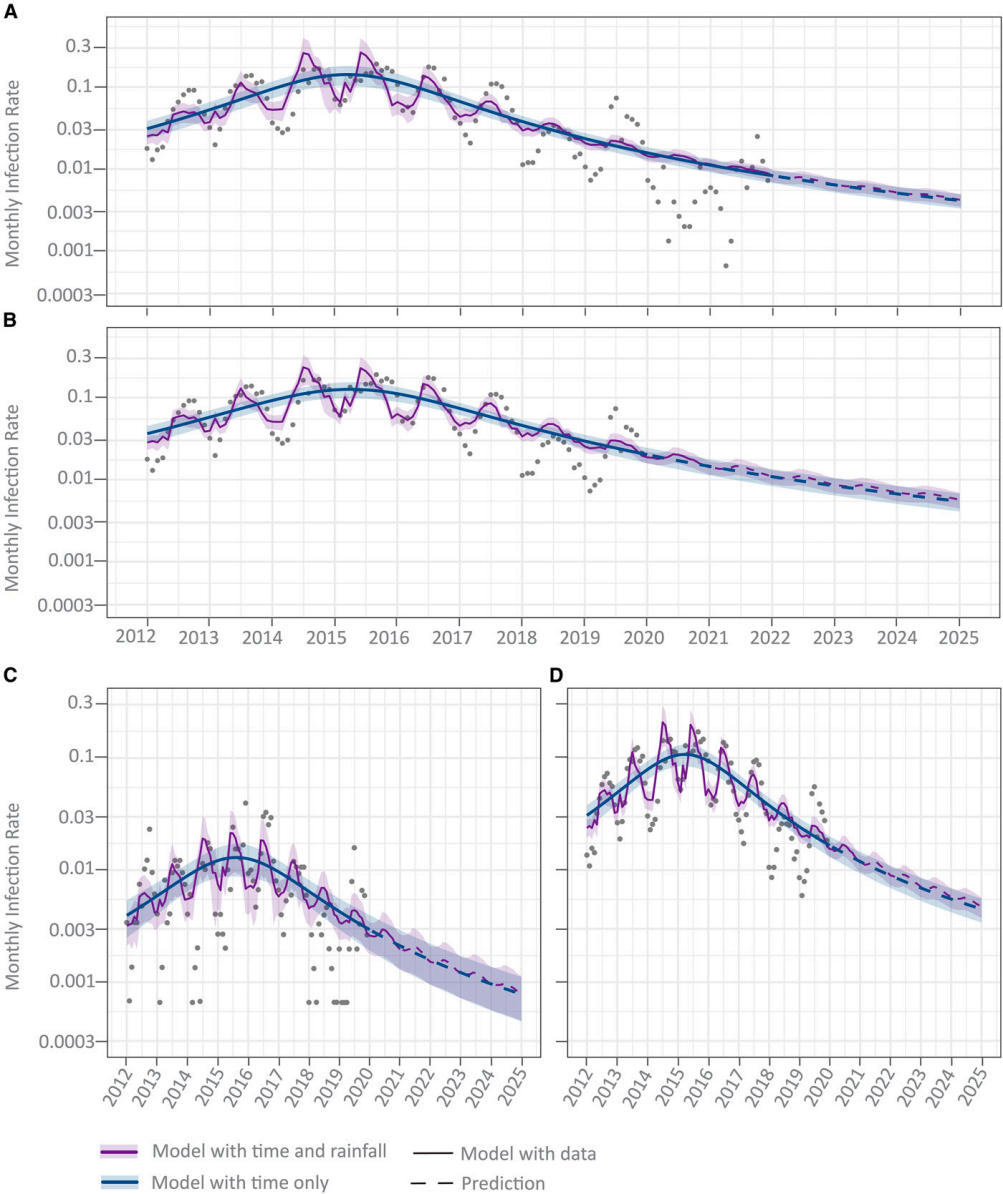
Cumulative monthly malaria transmission using the Model of Environmental Factors with inclusion (**A**) and exclusion (**B**) of data during the COVID-19 pandemic years (2020–2021) and *P. falciparum* (**C**) and *P. vivax* (**D**). The observed infection rates are overlayed on two prediction curves: The purple model includes the quadratic spline for time and a covariate for cumulative monthly rainfall, and the blue model contains only the quadratic spline for time. Solid lines represent predictions from the model with actual data, and the dashed line represents predictions outside of the observed data range. The colored ribbons for each model represent the 95% CI for infection. *P. falciparum* = *Plasmodium falciparum*; *P. vivax = Plasmodium vivax*.

We built two models of malaria transmission in Goa based on GMC case numbers and meteorological parameters of rainfall, humidity, and time. The first model incorporated all three factors into the model, and the second model generated the trend in malaria transmission using only time (Figure 6A; Supplemental Figure 5A). Environmental factors greatly improved the fit of the model to the observed data, whereas the model with just time demonstrated an apparent downward trend in monthly infection rates observed year over year. The number of malaria cases increased by 1.2% for every 100-mm increase in rainfall, by 0.05% for every 1°C rise in minimum temperature, and by 0.1% for every 10% rise in relative humidity. For every 1°C rise in maximum temperature, the probability of malaria cases decreased by 0.5%.

A clear trend of reduction in malaria transmission rate and cumulative malaria cases was evident in the yearly pattern (Figure 6A; Supplemental Figure 5A). The projected data suggest that if the current trend continues, malaria transmission in Goa at the beginning of 2025 will be almost insignificant, with 10 cases per 1 million residents (API: 0.0095, 95% CI: 0.075–0.0114). The model was limited using time as a proxy for unaccounted data such as demographic shifts (population mobility, imported cases), bed net usage, policy changes, and reservoir changes that likely contributed to this decline. Because data on these unaccounted variables are not available in the public domain and we could not determine them ourselves, these could not be analyzed as variables along with the GMC malaria case counts and meteorological factors. The cubic spline of calendar time variable represents the unaccounted data as a stand-in variable. Further, because the data were fit using a 2-degree spline, the model with just time approximates a quadratic equation; thus predictions go asymptotically to zero as we predict time farther out from the collected data.

We posited that restrictions related to the COVID-19 pandemic from March 2020 might have influenced health-seeking behavior (Figure 1E) and data reporting. Furthermore, a pause in outdoor construction activities during this period meant that there were far fewer construction workers present, a demographic that accounts for a sizable proportion of the transmission reservoir in the GMC catchment region. We built a variation of the model to check if COVID-19–related disruptions affected the overall trend of transmission. A slightly less steep downward trend and a projected API of 0.005 by 2025 was observed when the model was trained with data from 2012 to 2019, excluding pandemic year 2020 (Figure 6B; Supplemental Figure 5B).

A species-specific version of the model showed similar downward trends for both *P. falciparum* and *P. vivax* transmission and cumulative cases (Figure 6C and D; Supplemental Figure 5C and D). However, the *P. falciparum* model indicated relative decoupling of malaria cases from rainfall compared with the *P. vivax* model.

## DISCUSSION

### Goa’s foray into the malaria elimination phase.

We analyzed the transitioning epidemiology of malaria in Goa with decreasing transmission intensity in the last decade, which has significant implications for malaria elimination in this state. After independence (1961), Goa experienced fluctuating malaria cases, reaching a peak of 25,975 in 1998^20^ after bringing annual reported case counts to zero in the first part of the 1980s and then again in 1992.^21^ As the largest tertiary health institution with a catchment region covering the entire state, the GMC has been able to capture the pattern of Goa malaria transmission reliably over the years.

As transmission intensity decreased, the epidemiology of malaria in Goa changed concurrently. *Plasmodium falciparum* malaria decreased more than *P. vivax* malaria, consistent with the knowledge that the latter is more resilient to interventions.^22^ The GMC observed a steady reduction in both uncomplicated and severe malaria cases, as well as malaria-related deaths, whereas health-seeking behavior and diagnostic activity remained unchanged. However, the proportion of severe malaria cases increased, possibly owing to declining protective immunity resulting from reduced exposure to the parasite,^23^ as observed in other low- to moderate-transmission regions in different parts of the world.^24^ The trend of decreasing average parasitemia and increasing average gametocyte density in response to decreasing transmission intensity suggests a shift toward submicroscopic infections^25^ and increased commitment to transmission.^18^

These indicators are important in gauging the impact of malaria control measures in the state, which involves using biological larvicides^26^ and providing long-lasting insecticidal nets to high-risk populations of migrant construction workers and local, below-poverty populations living close to construction sites.^27^^,^^28^ The Goa Public Health Act (1985)^29^ stipulates that the Directorate of Health Services should focus on limiting transmission in the high malaria–risk group of migrant construction workers by screening them for malaria on arrival in the state and issuing health cards for follow-ups, along with stringent case management at the PHC level so that each patient with suspected malaria is tested with RDT and promptly treated if malaria-positive.^27^^,^^28^ The GMC follows a similar policy, although malaria diagnosis is done only through smear tests. The GMC-based malaria transmission indicators, including absolute and relative numbers of malaria cases as well as SPR, suggest that the malaria burden of the state has been steadily decreasing from 2018 onward to the elimination phase level.^5^^,^^28^ Projections from our transmission model suggest that in a “business as usual scenario” (no malaria control service disruption),^30^ transmission in Goa will be almost negligible by 2025.

### Implications of seasonal differences in Goa malaria cases.

Identifying location-specific seasonal patterns derived from monthly malaria data and regional meteorological parameters helps to determine whether malaria transmission is perennial and endemic, as in the high-rainfall region of Goa,^31^ or variable and epidemic prone, as in the arid and semiarid regions of northwest India.^32^ This is useful for planning interventions and developing early warning systems through seasonal forecasting. For example, epidemic outbreaks can occur because of environmental deviations such as prolonged rainfall in regions with strongly seasonal malaria and unstable transmission, such as semiarid regions of northwest India.^33^ Also, a standardized definition of seasonality, similar to ours, can be made comparable across locations to facilitate seasonal transmission modeling and support policy decisions.

### Role of climate and socio-demography in Goa malaria transmission.

Our environmental model of transmission evaluated the role of rainfall, temperature, and relative humidity on malaria transmission in the GMC catchment region, establishing a validated relationship between meteorological parameters and epidemiology. We noted that cumulative monthly rainfall was a significant predictor of total and *P. vivax* seasonal malaria but not of *P. falciparum* seasonal malaria. Monthly average relative humidity and rainfall were highly correlated (Spearman ρ = 0.84) in Goa, which means these factors can be considered somewhat interchangeable in this region. Although rainfall and temperature are staple parameters for malaria transmission models and reliable indicators of transmission in India,^34^^,^^35^ relative humidity has been recently reported as an important driver of urban transmission by *An. stephensi*.^36^

Males in the 15–50-year-old group had the highest proportion of malaria cases in Goa, consistent with other parts of India,^37^^–^^40^ whereas the above-50 group was the highest contributor to transmission in terms of gametocyte density and proportion of gametocyte-positive samples. The majority of Goa’s population (90%) lives in permanent, well-built houses with electricity.^41^ Fifty-nine percent of Goa’s population lives in urban areas.^41^ Ninety percent of malaria patients treated in the GMC were also from these areas. Lower socioeconomic groups; people with occupations that expose them to mosquito bites, such as night guards; residents living at the periphery of construction sites; and mobile populations are some of the groups at highest risk of malaria in urban areas.^42^ The socio-demography of MESA-ICEMR–enrolled malaria patients fits this broad pattern, with construction workers constituting the single largest group within the infected population and a little over one-tenth of the infected population having traveled to Goa within the last month. Construction workers accounted for more than 50% of *P. falciparum* malaria patients during the NMAL and might be acting as an infectious reservoir during the dry season. However, the proportions of construction workers who were *P. vivax* patients during the NMAL and MAL were similar. Nevertheless, this pattern suggests that temporary residences used by construction workers can benefit from spraying during both dry and wet seasons.

### Malaria hotspots.

Malaria hotspots in the elimination scenario of Goa are “areas of high burden that may serve as a source to maintain transmission.”^43^ Malaria transmission in the GMC catchment region is heterogeneous but focal^6^ around the populous urban cities of Panjim, Mapusa, Vasco, Verna, and Margao and is consistent with other low-transmission regions in the elimination phase.^44^^,^^45^ This heterogeneous transmission in the designated urban areas^46^ of the GMC catchment region could be partially explained by a lower entomological inoculation rate in city centers compared with peri-urban and rural regions.^47^

During the study period, the Panjim, Chimbel, and Cortalim PHC regions in the GMC adjacent area (Supplemental Figure 4A) saw extensive construction activity. We believe that these activities contributed to a significant proportion of malaria cases treated at the GMC, as evidenced by the demographic composition of construction workers in MESA-ICEMR–enrolled cases. A subset of these construction workers, who had traveled to Goa within the last month, were found to be clustered in the Panjim and Chimbel PHC regions. Detailed molecular analysis of this subset in the future might hint if their infections were acquired outside Goa or were locally contracted. This will help in assessing imported malaria risk and strategizing to minimize local malaria transmission around imported infections.

### Translational value and regional impact.

In this study, we report the ecological receptivity, population vulnerability, and epidemiological elements of malaria transmission in Goa to stratify malaria cases, which could inform cost-effective and targeted interventions tailored to local contexts.

Although construction workers formed the single largest demographic group at risk, tailoring malaria control strategies to only this group may have limitations, as Goa has several transmission-receptive areas and vectors that are capable of carrying on local transmission.^21^ Continued integrated vector management at all transmission-receptive areas in this state might be effective in preventing a resurgence. Local transmission in Goa could move toward more submicroscopic infections, as seen in other low transmission–intensity regions.^25^ This means residual malaria transmission can be harbored in the local population with parasite numbers below detection level and no visible clinical symptoms for the disease. Goa will need a mass screening strategy to detect and treat asymptomatic cases and address this issue.

Goa has experienced rapid urbanization in the past decade,^48^ with 35% growth in urban population.^49^ Rapid urbanization and a construction boom in this state mean that there are potentially more unsupervised manmade water storage facilities, which could provide ideal breeding grounds for Anophelines.^50^^,^^51^ A warm, humid climate with heavy rainfall during the monsoons also provides ideal environmental conditions for vector growth and malaria parasite development within the vector. Local vector *An. stephensi*, being a highly specialized urban vector, can thrive under these conditions. Goa provides an interesting socioeconomic background for studying malaria transmission. This smallest, most affluent^52^ state in India with a relatively high literacy rate (88%)^52^ and healthcare accessibility^53^ is also a tourist hub with a high incoming migration rate.^54^ Heterogeneous socioeconomic conditions such as those between the relatively well-settled local population and the temporary accommodations of migrant construction workers add to the disproportionate malaria risk. Spatial risk of urban malaria cases stratified by population density and incoming migration could report different public health burdens in different regions and inform the urban malaria scheme^55^ of the NMCP.

Our study provides the first comprehensive, statewide, 10-year malaria transmission data stratification at village-level resolution and transmission model in Goa. A similar health facility–based survey incorporated with geolocation in a low-transmission setting reported comparable results between health facility and community-based estimates of infection,^56^ which, along with other published reports,^57^^–^^62^ indicates the suitability of health facility–based surveillance as a proxy for region-wide traits. However, our study has some limitations. Only care-seeking, febrile individuals were screened, which means non–care-seeking individuals were overlooked and we missed potential submicroscopic/asymptomatic infections. We did not have the resources to do a case control study and hence could not evaluate the risk of contracting malaria stratified by different parameters. Because 80% of the GMC malaria cases resided in the predominantly urban neighboring regions, it is possible that this study overestimates the proportion of urban malaria cases in Goa. Also, data related to specific malaria control strategies and their impact are missing from our transmission model owing to the nonavailability of this information in the public domain. So, caution is required when using this health facility–based model for assessing the impact of control programs.^63^

Goa previously went through elimination phases before malaria cases rebounded.^21^ Although we will be cautiously monitoring this elimination phase, our study offers insights into the possible endgame of malaria transmission in Goa.

## Supplemental Materials

10.4269/ajtmh.23-0828 Supplemental Materials
