# Supplementary material for: Descriptive, Hospital-Based, 10-Year Study of Malaria Transmission in Goa, a Southwest Indian State in the Malaria Elimination Phase
Source: Am J Trop Med Hyg. 2024 May 7;111(1):11–25. doi: 10.4269/ajtmh.23-0828 (PMC11229636; doi:10.4269/ajtmh.23-0828)
Supplement: Supplemental Materials [file tpmd230828.SD1.pdf]

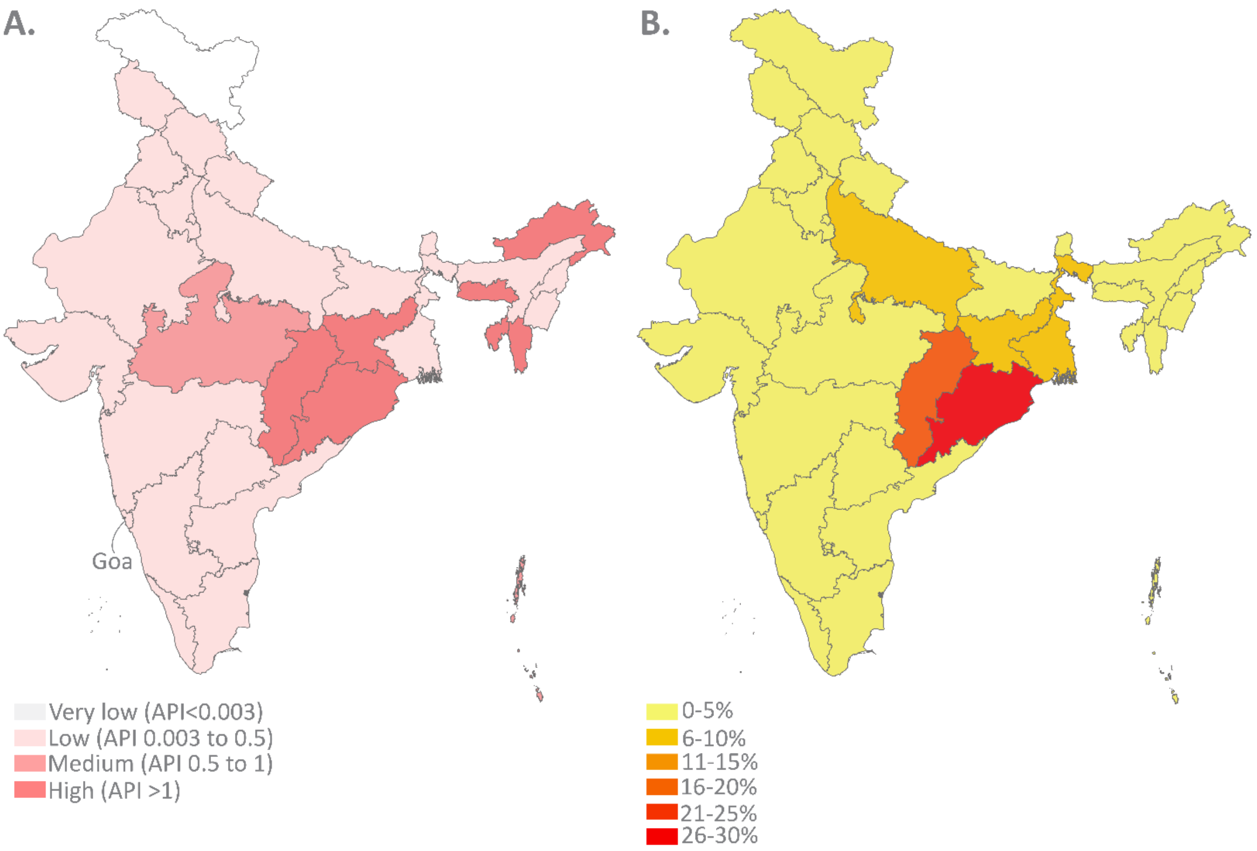

Supplementary Figure 1: Malaria transmission intensity and load in Goa with respect to transmission in the rest of India. A.) Transmission intensity represented as the WHO pattern transmission continuum. B.) Transmission load. Goa ranks 12 in terms of Annual Parasitic Index (API) and 23 in terms of transmission load among 36 Indian states.

API and transmission load was derived from published malaria case numbers by National Center for Vector Borne Disease Control (https://nvbdcp.gov.in/index1.php?lang=1&level=1&sublinkid=5784&lid=3689).

The transmission continuum in India is modeled with the WHO API based template (https://www.who.int/malaria/publications/atoz/WHO-malaria-elimination-framework-2017-presentation-en.pdf)

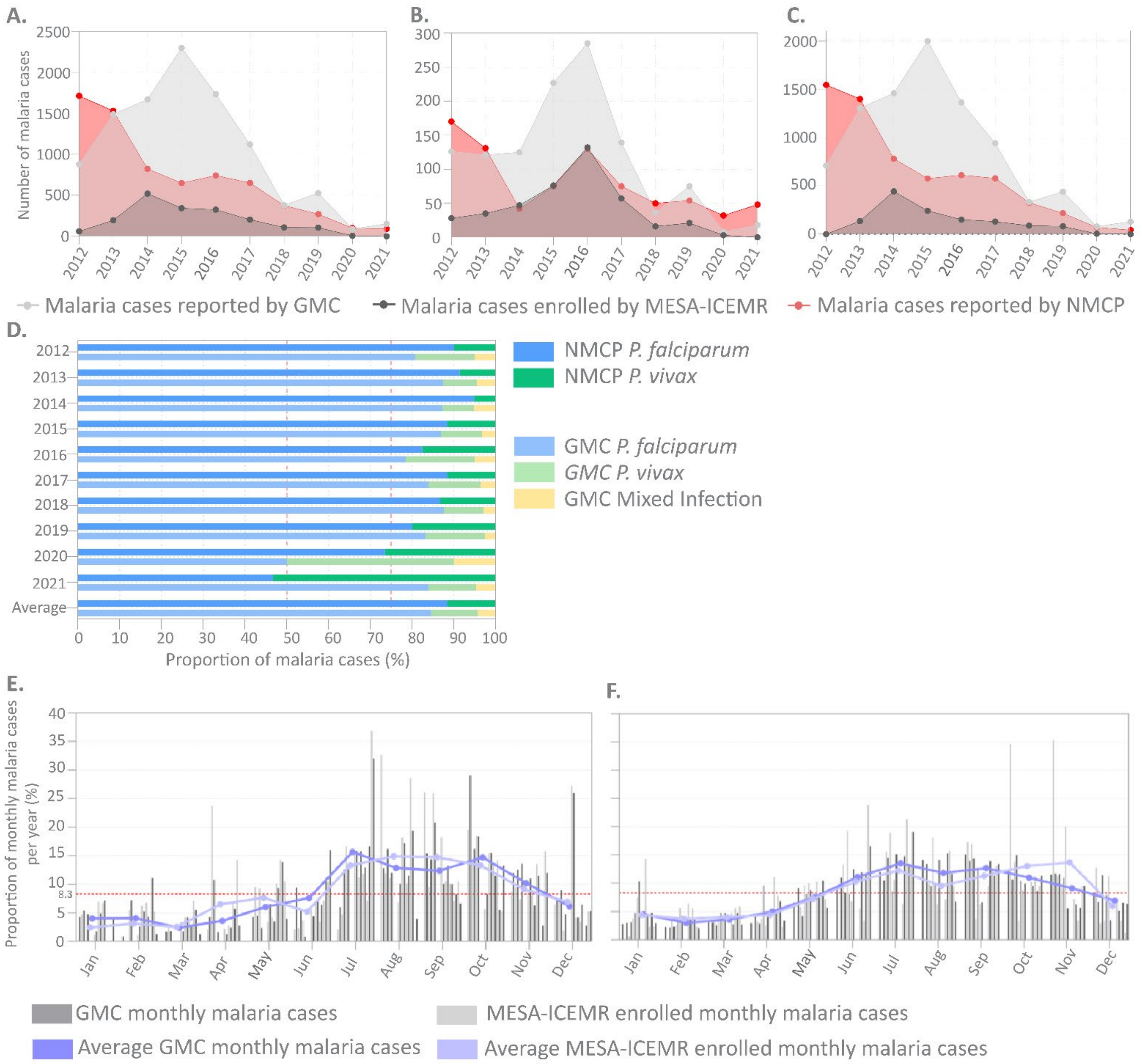

Supplementary Figure 2: Validation of using total GMC malaria cases as a subset of total Goa malaria cases in the context of A.) total malaria cases B.) *P. falciparum* cases C.) *P. vivax* cases and D.) *Pf*: *Pv* ratio. Validation of using MESA-ICEMR enrolled E.) *P. falciparum* and F.) *P. vivax* cases as a subset of total GMC cases. The red dotted line denotes the average yearly proportion of malaria cases (100/12=8.3%) to demarcate seasonal transmission.

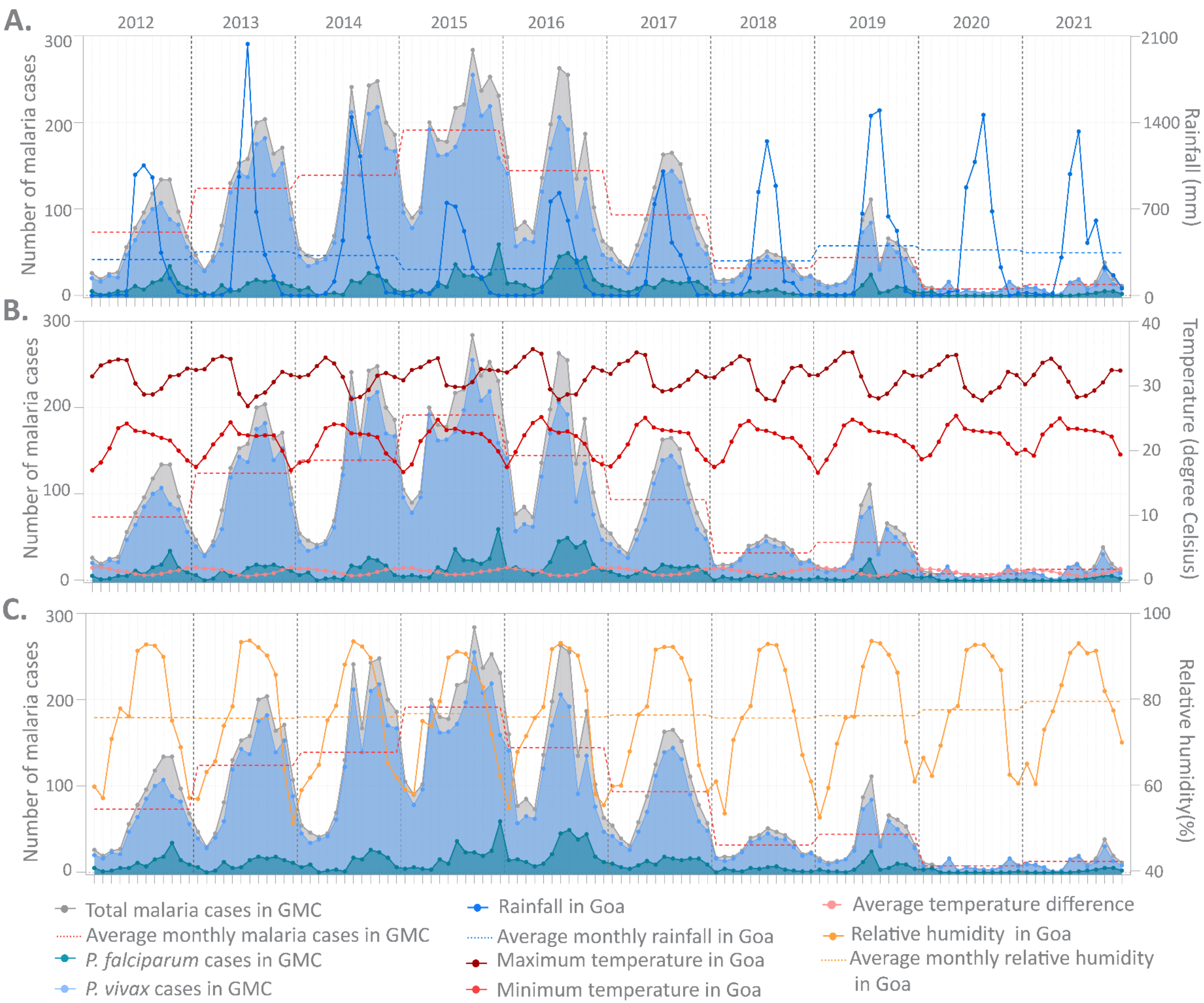

Supplementary Figure 3: Interannual variation of GMC malaria cases relative to A.) rainfall B.) temperature and diurnal variation of temperature, and C.) relative humidity.

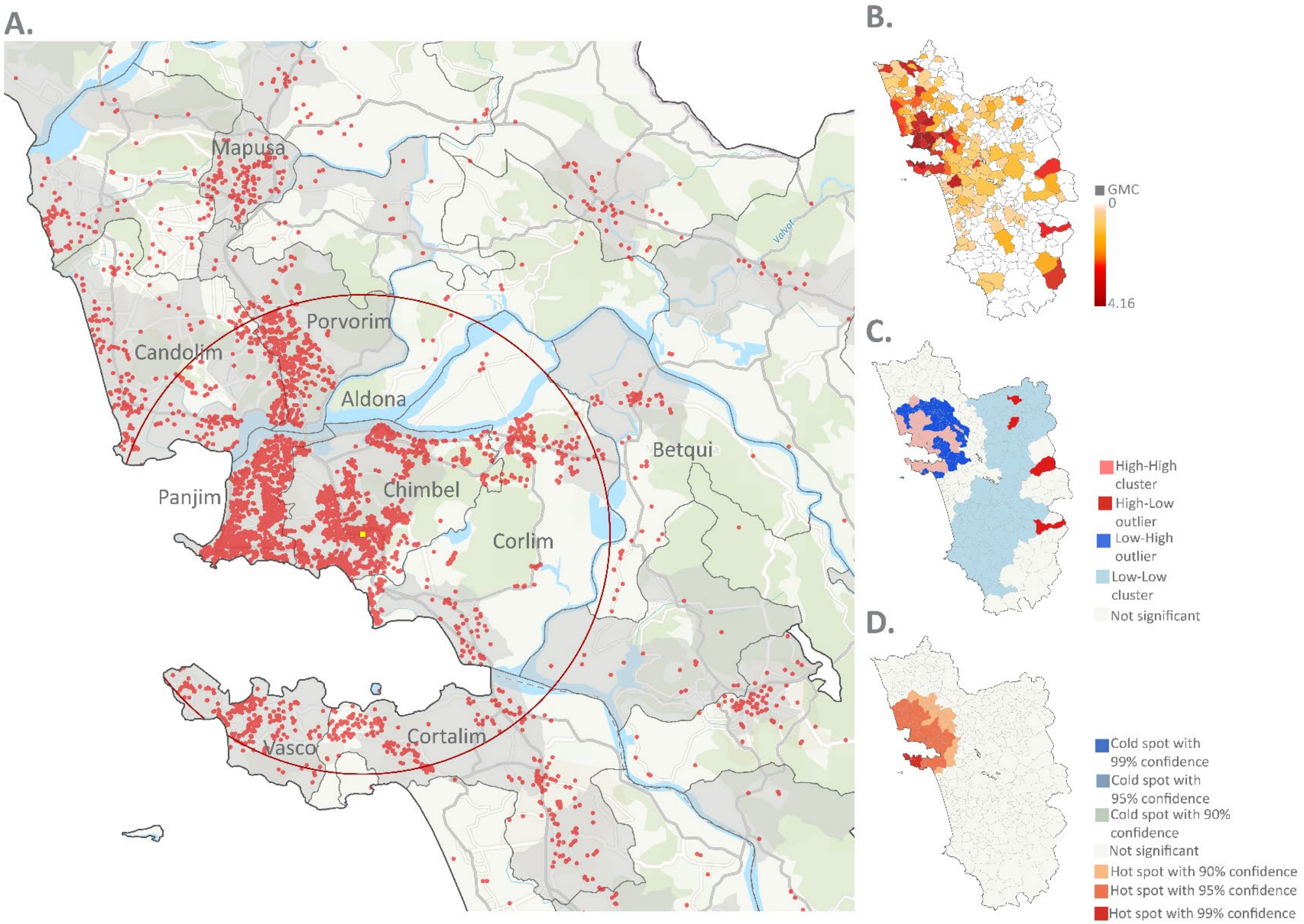

Supplementary Figure 4: Hotspot for malaria cases treated by GMC. A.) About 80% of the infected cases treated in GMC (in the circle) are from the neighboring, mostly urban PHCs (labelled). Internal map boundaries represent PHC boundaries. B.) Spatial stratification, C.) Anselin Moran's Local I analysis and D.) Getis Ord GI* analysis of GMC malaria patients, adjusted by population (API).

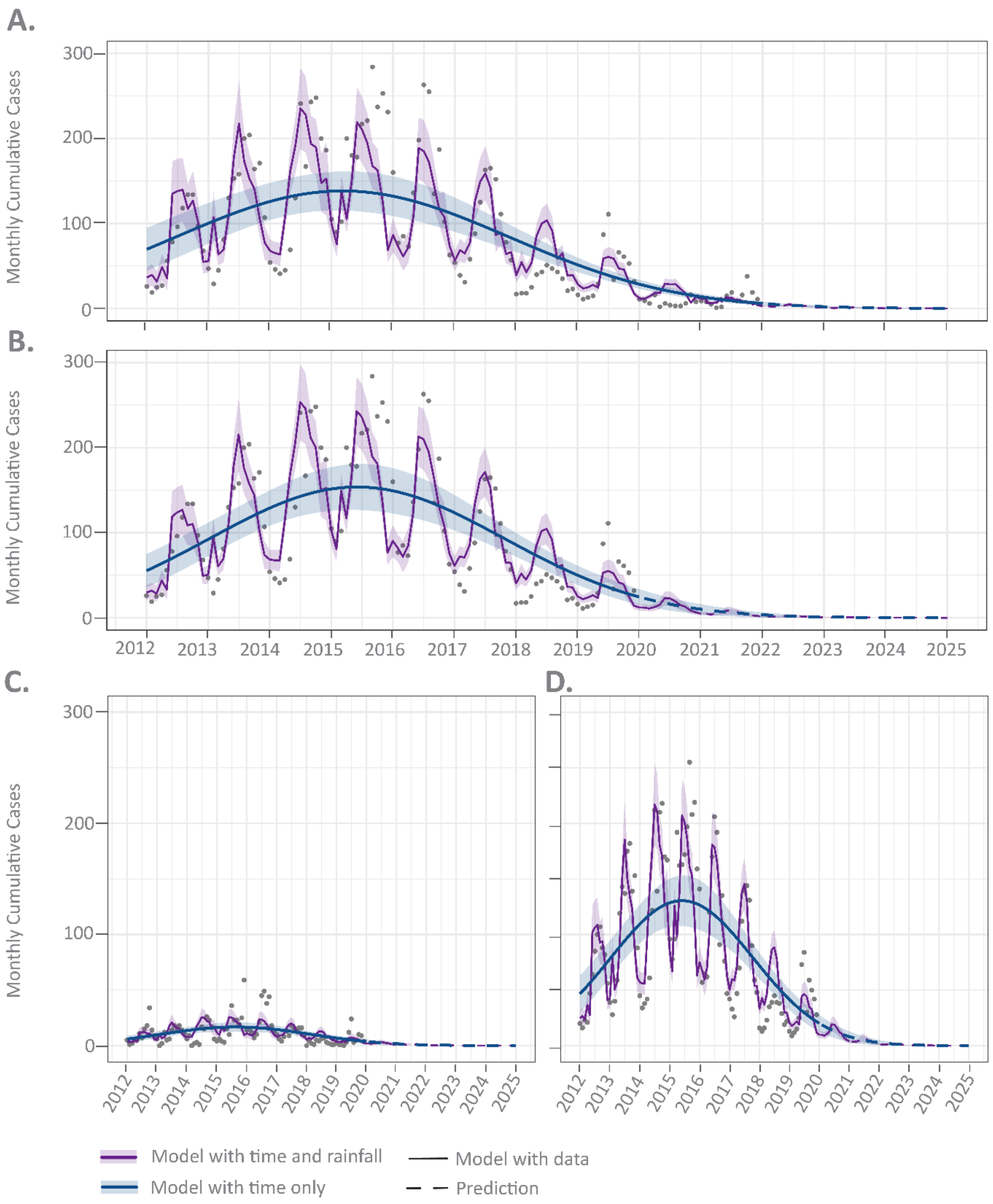

Supplementary Figure 5: Cumulative Monthly Malaria Cases using the Model of Environmental Factors with A.) inclusion and B.) exclusion of data during the Covid-19 pandemic years (2020-2021), and of C.) *P. falciparum* and D.) *P. vivax*. The observed cumulative cases are overlayed on two prediction curves: (purple) model includes the quadratic spline for time and a covariate for cumulative monthly rainfall, and

(blue) contains only the quadratic spline for time. Solid lines represent predictions from the model with actual data, and the dashed line represents predictions outside of the observed data range. The colored ribbons for each model represent the 95% confidence interval (CI) for cumulative cases.

A. Malaria Transmission outcome indicators stratified by year	2012	2013	2014	2015	2016	2017	2018	2019	2020	2021
**1. Disease**										
**1.1. GMC**										
1.1.1 Confirmed malaria cases										
a. Number	878	1489	1670	2298	1734	1121	381	528	90	152
b. Proportion among all patients treated at GMC (%)	0.17	0.28	0.32	0.44	0.33	0.21	0.07	0.10	0.02	0.03
1.1.2. Inpatient malaria cases (severe malaria)										
a. Number	147	172	217	266	337	317	123	185	52	67
b. Proportion among all inpatients treated at GMC (%)	0.25	2.46	0.34	0.40	0.49	0.45	0.17	0.24	0.09	0.10
c. Proportion among all malaria patients treated at GMC (%)	17	12	13	12	19	28	32	35	58	44
1.1.2.1 Inpatient malaria cases (severe malaria) *P. falciparum*										
a. Number	34	34	38	60	129	80	27	35	9	11
b. Proportion among all inpatients treated at GMC (%)	0.06	0.06	0.06	0.09	0.19	0.11	0.04	0.05	0.01	0.02
c. Proportion among all malaria patients treated at GMC (%)	3.9	2.3	2.3	2.6	7.4	7.1	7.1	6.6	10.0	7.2
d. Proportion among all *P. falciparum* patients treated at GMC (%)	26.98	27.87	30.40	26.43	45.26	57.55	75	46.67	100	18
1.1.2.2 Inpatient malaria cases (severe malaria) *P. vivax*										
a. Number	99	130	156	187	163	210	89	142	42	53
b. Proportion among all inpatients treated at GMC (%)	0.17	0.22	0.24	0.28	0.24	0.30	0.13	0.19	0.07	0.08
c. Proportion among all malaria patients treated at GMC (%)	11.3	8.7	9.3	8.1	9.4	18.7	23.4	26.9	46.7	34.9
d. Proportion among all *P. vivax* patients treated at GMC (%)	13.96	9.98	10.70	9.36	11.97	22.32	26.65	32.27	53.16	40.46
1.1.2.3 Inpatient malaria cases (severe malaria) mixed infections										
a. Number	14	8	23	19	45	27	7	8	1	3
b. Proportion among all inpatients treated at GMC (%)	0.02	0.01	0.04	0.03	0.07	0.04	0.01	0.01	0.002	0.004
c. Proportion among all malaria patients treated at GMC (%)	1.59	0.54	1.38	0.83	2.60	2.41	1.84	1.52	1.11	1.97
d. Proportion among all mixed patients treated at GMC (%)	32.56	12.12	26.44	25.68	51.72	65.85	63.64	61.54	16.67	42.86
1.1.3. Blood smears examined (BSE)	15595	16908	19454	22444	21850	20579	16037	21226	10867	13136
1.1.4. Malaria test positivity rate (SPR) (%)	5.6	8.8	8.6	10.2	7.9	5.4	2.4	2.5	0.8	1.2
1.1.5. Percentage of malaria cases due to *P. falciparum* (%)	14.4	8.2	7.5	9.9	16.4	12.4	9.5	14.2	9.6	11.0
1.1.6. Percentage of malaria inpatient cases (%)	0.0	0.0	0.0	0.1	0.1	0.1	0.0	0.0	0.0	0.0
1.1.7. Annual Blood Examination Rate	1.1	1.1	1.3	1.5	1.5	1.4	1.1	1.4	0.7	0.9

1.1.8. Population at risk	1.46	1.47	1.48	1.48	1.49	1.50	1.50	1.51	1.52	1.52
1.1.9. Estimated malaria incidence (API)	0.60	1.01	1.13	1.54	1.16	0.75	0.25	0.35	0.06	0.10
1.1.10. Prevalence (%)	5.6	8.8	8.6	10.2	7.9	5.4	2.4	2.5	0.8	1.2
**1.2. MESA-ICEMR enrolled**										
1.2.1. Mean age (years) for clinical malaria	32.0	26.1	27.6	26.9	28.0	29.6	29.6	29.3	ND	ND
1.2.2. Mean age (years) for severe malaria	33.0	31.9	28.4	28.9	31.1	32.6	30.5	29.4	ND	ND
1.2.3. Incidence of cerebral malaria (per 100)	9.7	1.0	1.2	0.3	2.2	2.9	0.0	0.0	ND	ND
1.2.4. Incidence of malaria associated anemia (≤11g/dl) (per 100)	30.6	6.1	6.5	9.9	16.0	25.9	17.4	17.6	ND	ND
1.2.5. Incidence of severe malarial anemia (Hb≤5gm dl) (per 100)	3.2	0.0	0.2	0.0	0.3	0.5	0.0	0.0	ND	ND
**2. Infection**										
**2.1. MESA-ICEMR enrolled**										
2.1.1. Mean parasitemia	2.06	1.86	0.57	0.49	0.59	0.47	0.09	0.35	ND	ND
2.1.2 Mean parasite density	11475.4	5506.1	5524.1	8962.3	7847	6270.7	11962.3	10023.6	ND	ND
2.1.3. Mean gametocytemia	0.04	0.089	0.024	0.009	0.0048	0.011	0.01	0.012	ND	ND
2.1.4. Mean gametocyte density	689.06	145.8	225.29	154.44	112.2	192.19	346.45	385.27	ND	ND
2.1.5. Mean parasite growth rate during adaptation	4.62	4.18	3.7	4.74	5.44	ND	ND	ND	ND	ND

**B. Malaria Transmission outcome indicators stratified by species**	***P. falciparum***	***P. vivax***	**Mixed infection**
1. Number of cases	8683	1145	429
2. Percentage of malaria caseload	85%	11%	4%
3 Transmission duration	Perennial	Perennial	Perennial
4. Transmission peak	July and September	July and October	July and October
5. Seasonal transmission duration (>yearly average)	June to November	July to December	July to December
6. Percentage change in case numbers since 2012	83 % decrease	88 % decrease	86 % decrease
7. Number (percentage) of severe cases	1267 (14.5 %)	457 (39.4 %)	155 (35.7 %)
8. Severe cases transmission duration	Perennial	Perennial	Perennial
9. Severe cases transmission peak	July and August	August and October	July and October

Supplementary Table 1: GMC based malaria transmission outcome indicators in Goa stratified by year and parasite species. API= Annual Parasite Index, ND= Not Determined

Age (years)	Gametocyte positive samples (by LM)		Average parasitemia (%)	Average parasite density (per µl)	Average gametocytemia (%)	Average gametocyte density (per µl)
	n	%				
**<5**	5	31.25	0.21	5378	0.002	128.87
**5-15**	13	28.26	0.49	7838.0	0.017	167.14
**15-50**	501	32.07	0.62	6989.1	0.016	202.32
**>50**	68	62.39	0.6	7753	0.02	252.65

Supplementary Table 2: Transmission reservoir stratified by age. LM= Light Microscopy

	MAL						NMAL					
	P. falciparum		*P. vivax*		Mixed		*P. falciparum*		*P. vivax*		Mixed infection	
	n	%	n	%	n	%	n	%	n	%	n	%
**Construction Worker**	131	39.6	466	52.2	22	52.4	68	51.1	175	47.9	14	46.7
**Other**	118	35.6	281	31.5	11	26.2	38	28.6	132	36.2	11	36.7
**Student**	28	8.5	35	3.9	2	4.8	7	5.3	19	5.2	2	6.7
**Not Employed**	17	5.1	24	2.7	4	9.5	3	2.3	5	1.4	1	3.3
**Housewife**	14	4.2	27	3.0	0	0.0	8	6.0	16	4.4	2	6.7
**Local Trader**	11	3.3	7	0.8	1	2.4	2	1.5	4	1.1	0	0.0
**Police**	4	1.2	25	2.8	1	2.4	3	2.3	8	2.2	0	0.0
**Factory Worker**	3	0.9	21	2.4	1	2.4	1	0.8	2	0.5	0	0.0
**Farmer**	2	0.6	0	0.0	0	0.0	1	0.8	1	0.3	0	0.0
**Plantation Worker**	2	0.6	5	0.6	0	0.0	1	0.8	3	0.8	0	0.0
**Miner**	1	0.3	0	0.0	0	0.0	0	0.0	0	0.0	0	0.0
**Forest Worker**	0	0.0	1	0.1	0	0.0	1	0.8	0	0.0	0	0.0
**Office Worker**	0	0.0	0	0.0	0	0.0	0	0.0	0	0.0	0	0.0
**Security**	0	0.0	0	0.0	0	0.0	0	0.0	0	0.0	0	0.0
**Soldier**	0	0.0	1	0.1	0	0.0	0	0.0	0	0.0	0	0.0
**Total**	331	100.0	893	100.0	42	100.0	133	100.0	365	100.0	30	100.0

Supplementary Table 3: Season and species-specific transmission reservoir stratified by occupation.
